# Normalizing fentanyl: interpreting the perceived ‘risk’ of correctional officer work

**DOI:** 10.1186/s13722-024-00504-3

**Published:** 2024-09-29

**Authors:** Rosemary Ricciardelli, Matthew S. Johnston, Gillian Foley

**Affiliations:** Fisheries and Marine Institute, Memorial University of Newfoundland, W3023, 155 Ridge Road, St. John’s, NL A1C 5R3 Canada

**Keywords:** Opioids, Overdose, Naloxone, Occupational stressors, Misinformation

## Abstract

**Background:**

Scholarship on how fentanyl affects the complexities of correctional settings is limited in Canada, as scholars have focused on the prevalence of opioid use and overdose in prisons, as well as community treatment and access following release. Fentanyl constitutes a continuing challenge both in prisons and broader society.

**Results:**

The current qualitative, interview-based empirical study examines how fentanyl is interpreted by correctional officers (COs, *n* = 99) across federal prisons in Canada, some of whom have worked in institutions with a high presence of fentanyl, while others have less exposure to the drug. We found that while many COs had responded to an overdose during their first or second year on the job, most COs who had did not perceive the event to be psychologically traumatic nor were concerned about the presence and availability fentanyl in their work environment, or they were indifferent. Yet this finding competes with the 41.4% of officers who did express concern about the presence of fentanyl – suggesting both a “normalization” of fentanyl as a workplace hazard as well as an underpinning social concern.

**Conclusions:**

We discuss the implications of these complicated findings in relation to reducing workplace stressors and countering misinformation that, in addition to other potential occupational factors, may be responsible for the concerns of COs tied to the presence of fentanyl.

## Introduction

The current empirical study examines how fentanyl is interpreted by correctional officers (COs; *n* = 99) across federal prisons in Canada, some of whom have worked in institutions with the presence of fentanyl, while others had more limited experience with the drug. More specifically, we unpack to what extent officers have scientific knowledge of the risks of fentanyl exposure and how perceptions of risk add to the occupational stress experienced by officers. Fentanyl, including derivatives like carfentanil, have heightened substance use concerns in Canada, with overdoses striking many urban and rural areas across the country [1]. Scholarship on how fentanyl affects the complexities of correctional settings is limited in Canada, as scholars have focused on the prevalence of opioid use and overdose in prisons during the public health emergency [2, 3], opioid treatment [4–6], as well as community overdose treatment services and accessibility following release [7]. Information on health and safety risks and perceptions of risks of fentanyl has also contributed to misinformation. For instance, Beletsky et al. conducted a study of how misinformation about health risks from casual contact with fentanyl spreads on social media. In 551 news articles spanning across 48 American States, misinformed media reports received approximately 450,000 Facebook shares, reaching nearly 70,000,000 users from 2015 to 2019 [8].

Scholarship consistently finds incarcerated men and women have higher rates of lifetime substance misuse and injecting substance use than the general population, with rates being most pronounced among incarcerated women [9]. Despite an individual’s gender, research indicates substance misuse often continues during incarceration because of the nature of addiction; the deprivations and boredom that usually accompany incarceration; other precipitating factors such as exposure to childhood trauma; and to build relationships within the prison setting [10, 11]. One study from Ontario, Canada reported over 56% of incarcerated people having disclosed using opioids, cocaine, crack, or methamphetamine in the year prior to their incarceration [12], which compares disproportionately to the estimated 21% of the Canadian population (approximately 6 million people) who will meet the criteria for addiction or problematic substance use in their lifetime [13]. The federal government of Canada has reported more recently that up to 80% of people incarcerated in federal penitentiaries have a drug or alcohol dependence problem [14]. Research focused on the context of women’s prisons suggests patterns of substance misuse may result from women’s efforts to self-medicate from previous traumas or other mental health problems, or to cope with the negative effects of incarceration such as social isolation and family breakdown [15]. Such factors could logically apply to men, women, and individuals who are gender diverse.

In the current study, we question how and to what degree fentanyl presence and/or exposure constitutes an operational stressor by answering the following research questions: (1) How do COs feel about fentanyl and their potential or actual exposure to fentanyl through their contact with incarcerated people?; (2) What concerns, if any, do they have about the drug’s presence and availability in the prisons in which they work? (i.e., do they have accurate scientific knowledge about the low risks associated with fentanyl exposure?); and (3) What factors shape COs’ perspectives on fentanyl in prisons, including their occupational responsibilities to respond to overdoses? (i.e., how does fentanyl inform occupational stress?). Underpinning each research question is the role misinformation plays in informing concerns; thus, we discuss the current climate around fentanyl in federal prisons and present considerations to counter misinformation.

## Literature review

In Canada, opioid use and availability impact all regions of the country, with the most affected regions being the provinces of Alberta and British Columbia [1]. Opioids such as oxycodone have tapered out as a result of drug-specific interventions, but fentanyl use has been on the rise [16, 17]. While other illicit drug overdose deaths have remained stable, fentanyl-related deaths appear to increase annually [1]. In Vancouver, British Columbia, fentanyl-users appear far more likely to experience an overdose in comparison to other drug users [18]. The increase in access to fentanyl is because of the high availability of both prescription and criminalized fentanyl products in the country [19]. The relevance here is how what happens in society is often imported and reflected in prison living.

In addition, fentanyl-related deaths increased as opioid users are shifting to illicit opioids [20]. Non-fentanyl-users too are unintentionally becoming exposed to fentanyl, with women being twice as likely to be unintentionally exposed compared to men [21]. Despite the implementation of fentanyl knowledge and awareness campaigns, most substance-users do not change their behaviours, believing their personal risk for overdosing is low [22, 23].

Fentanyl is also tied to increases in overdoses among users in prisons and in society and thus impacts all people in correctional settings [1], which is not surprising given prisons are reflections of broader, free society [24]. Yet, while media coverage on the problem of fentanyl was, for a time, widely circulated and critically discussed [25], in Canada, at least, the presence of fentanyl was, like most things, sidestepped by the COVID-19 pandemic/endemic—a phenomenon still acquiring attention but less extensively given the impact of the unprecedented global pandemic. This is concerning as fentanyl still constituted a challenge during the pandemic for all [26], and thus, in prisons specifically, placed additional occupational strain on COs who were obligated to respond to its presence – a context underpinning the data in the current study which were collected during the pandemic.

Although many incarcerated people who use drugs did so prior to incarceration [27, 28], some incarcerated people initiate substance use once in prison [29]. Despite differences in substance use patterns based on a variety of risk factors (e.g., prison security level, length of incarceration, gender), substance use and access tends to prevail regardless of the form of prison governance or socio-demographic characteristics of the institution [28, 30, 31]. Moreover, little is known about the degree of, or lack of, evidence-informed treatments offered in prisons.

Research supports the conclusion that people working and living in prisons fear fentanyl being mixed with other substances and being used against them, and are aware that the presence of fentanyl increases their likelihood of an overdose [32]. Most alarming about incarcerated people’s substance use is the three-fold increase in opioid-related overdoses in the Canadian federal prison system from 2012/2013 to 2018/2019 [3]. Further, fentanyl was the most common opioid involved in these critical incidents, increasing from 3% of all overdoses in 2012/2013 to 47% in 2017/2018. McKendy et al., in a study commissioned by Correctional Service Canada (CSC), report that, in the federal prison system, 92% of overdoses occur between 6 AM and midnight, and over half of overdoses (56%) involve opioids, with 23% of overdoses being attributed to fentanyl alone [3]. For example, between 2012 and 2019, there were 530 overdoses (75 per year on average or about 4 per 1,000 prisoners). In 53% of overdoses, officers applied naloxone (also referred to as Narcan), with 6% resulting in death and 15% being classified as intentional overdoses. According to CSC, the most common profile of incarcerated people overdosing is single, white (majority), age 25–34 years old, with less than a high school diploma; over half (54%) were incarcerated in a medium security facility; and a third of overdosing incarcerated people were sentenced to less than four years. Regarding their mental health, half of the overdosing prisoners had a diagnosis for mood disorder and/or anxiety disorder, and 86% suffered from substance misuse/addiction issues [3].

Beyond affecting prisoners, opioids are argued to pose a risk to correctional workers. Bucerius and Haggerty found COs fear they will be exposed to fentanyl either accidentally or intentionally [32]. While officers have been hospitalized due to fentanyl exposures [33, 34], these fears appear to be largely informed by scientifically unsupported statements, policies, or trainings circulating within institutions, (2) popularly shared misinformation, and/or (3) the misunderstanding of hospitalizations of officers due to fentanyl exposure (which may be due to a reactive/cautious response rather than being supported by toxicology that shows an actual overdose occurred versus exposure). For example, in their qualitative study of 23 United States law enforcement leaders and officers across five law enforcement agencies, Attaway et al. found nearly all participants interviewed wrongly believed dermal exposure to fentanyl was deadly and expressed fear about such exposure on scene [35]. These findings resulted from a lack of education about fentanyl exposure and sources of misinformation not based on rigorous scientific evidence. Given misinformation around fentanyl exposure is widespread among police in the United States, del Pozo et al.’s study found improved police education showed promising results in reducing false beliefs about fentanyl [36].

Nevertheless, a recent Canadian study suggests fears have resulted in increased vigilance among COs, including the need to wear personal protective equipment during responses to overdoses, which unfortunately does add to the response time and thus can affect the well-being and life of an overdosing incarcerated person [32]. Of note, however, Bucerius and Haggerty’s study was conducted in a Canadian province profoundly affected by fentanyl, in prisons with high prevalence of use and overdoses, at a time when the problem of fentanyl remained relatively acute [32].

To help prisoners who use opioid drugs, Canadian prisons have implemented opioid agonist treatment (OAT) programs similar to those in the community, dispensing both methadone [30, 37–39] and buprenorphine/naloxone [5, 37, 38, 40, 41]. Prisons now provide access to naloxone kits for overdoses [41, 42] – however this access is limited. Naloxone is not part of the first aid kit; as such, the life-saving medication is stored independently, not carried by officers generally, and often must be retrieved through contact with the nurse or supervisor on shift. Moreover, access to, training for, and administration of naloxone by COs is an essential service.

Challenges also arise as prior to the era of OAT, incarcerated people in some jurisdictions were forced to completely stop opioid usage without treatment or were forced to stop OAT as a punitive measure, resulting in many incarcerated people suffering from acute and protracted withdrawal; such a practice also places incarcerated people at a high risk for overdosing in the future due to a decrease in opioid tolerance [43–45]. This practice also contradicts research which continues to find that OATs in Canada have been successful in helping people during incarceration and upon release [4, 45]. Carroll et al. found in their study of 92 adults who use opioids in Providence, Rhode Island that many participants perceived long-term relationships with “trusted dealers” to be a key strategy for reducing the risk of substance use-related harm due to suppliers’ alleged adoption of consumer protections strategies (i.e., refusing to sell fentanyl, testing drugs for fentanyl before sale) [46]. This study demonstrates the importance of drug safety among some people who use drugs (and agency) who will resort to what safety practices are available to them in light of or in absence of adequate resources, education, or programming in their respective communities. This is necessary to emphasize in the current study, as upon re-entry, if there was no OAT continuity and program available, there is heightened overdose risk for participants (i.e., tolerance down, cravings continue, high stress during transition, dangerous supply) [46].

### Current study

Fentanyl and opioids more generally constitute an occupational stressor; however, little is known about how the real or perceived threat of fentanyl affects COs working in federal institutions. To this end, we question how COs feel about working in environments where fentanyl is present and the effects of this on their interpretations and stressors, which we argue are, at least in part, being shaped by misperceptions and/or misinformation about exposure to fentanyl.

## Method

Our analysis is based on semi-structured interviews with 99 COs from a larger multi-year, mixed-methods study (2018–2028) on the mental health and well-being of COs in Canada’s federal prison system [47]. Participants in our sample consist of 62 men and 37 women with the majority of participants aged 25–34 (52.5%). The remaining participants were aged 19–24 (29.3%), 35–44 (13.1%), and 45–54 (5.1%). Our sample included, in terms of race/ethnicity, white (76.8%), with the remaining participants identifying as a person of colour (13.1%) or Indigenous (8.1%; two participants did not report race/ethnicity). A high percentage of participants were single or never married (50.5%) or in a marital or in a common-law relationship (39.4%), with the remaining being either separated or divorced (7.1%). Participants had either a high-school diploma (28.3%) or education beyond a high-school diploma, completing either a college diploma, including trades and vocational training, some sort of non-university certificate (43.47%) or a university degree (25.3%). Three participants did not report their educational background. Approximately a quarter of participants (27.3%) had correctional work experience prior to joining CSC, which typically involved working in Canada’s provincial or territorial correctional systems. Three participants did not report whether they had prior correctional experience.

CO recruits were interviewed prior to starting the job (i.e., baseline interviews) and annually after that (i.e., follow-up wave interviews). The interviews included in the current study were conducted between October 2019 and March 2022 as part of the wave one follow-up interviews with officers following their first or second year on the job. The timing of year one follow-up interviews was affected by COVID-19 such that some one-year follow-up interviews were delayed by upwards of eight months due to the public health measures and research, accordingly, paused temporarily. The interviews used in the current analysis were included based on applicable content and availability, as each was transcribed, coded, and thus ready for data analysis and interpretation.

Interviews included in this analysis discussed fentanyl, with most data emerging from the question: “Does the presence of fentanyl affect perceptions of safety among staff?” We intended this question to start a discussion about officers’ thoughts about fentanyl, specifically if the presence of fentanyl adds to occupational stress, possibly harms wellness, or impacts responsibilities. We also asked a subset of 58 of the participants if they “had ever responded to an overdose”, to understand how the act affects them or is experienced. Overall, interviews used a semi-grounded conversational format [48, 49], the impetus being to determine if fentanyl contributes to the high rate of Operational Stress Injuries [50] among officers. CSC helped facilitate interviews, allowing interviewees to participate during paid work hours. Due to COVID-19, we conducted most interviews over the phone, each lasting between 45 and 90 min on average. Interview data were voice recorded and transcribed verbatim, with all information being anonymized and identification numbers applied to protect participant confidentiality. Despite CSC’s collaboration, participation was voluntary, and CSC has no access to primary research data or participant information [47].

Our research is inspired and guided by “appreciative inquiry,” which is an approach rooted in a position of appreciation, empathy, or participatory understanding of social phenomena, and driven by the point of view of participants [51]. Simply said, in the current study, we approached the data wanting to understand COs’ nuanced perspectives on fentanyl while recognizing the challenge employees have in trying to address occupational health and perceived risk. As such, while we do take up COs’ experiences critically, the explicit focus of the study is not to invalidate their perspectives or diverse meanings.

We coded interview transcripts thematically according to a multi-item coding scheme using NVivo software. This scheme included a category labeled “contraband,” a sub-category labeled “products,” and a further sub-category labeled “fentanyl” under which the excerpts supporting our analyses were coded. Next, using MS Excel, we analyzed the interview topic, applying a combination of open-coding [52] and axial-coding [53] to determine the following themes: “fentanyl and overdoses,” “concerned about fentanyl,” “indifferent to fentanyl,” “impacts,” “cell searches, personal protective equipment, and institutional security,” and “naloxone.” Finally, we used Jamovi statistical software to tabulate participant demographics and examine the data for patterns and associations involving demographics and participants’ concern or indifference to fentanyl. The research’s ethics protocols received approval from the Research Ethics Board of Memorial University of Newfoundland (File No. 20190481).

With our sample characteristics in mind, we ground our analysis in the context in which officers work. Beyond the presence of fentanyl, which can be ambiguous (and thus contribute to misinformation), many factors can create stress for COs, some being organizational in nature (i.e., shift work, colleagues, management) and others operational (i.e., inherent to the job, such as responding to an overdose). Although beyond the scope of the current article to unravel in its full complexity, we emphasize that these contextual factors are also at play and must be recognized in interpreting our results.

## Results

In our sample of 99 COs with a maximum of two years’ experience working in a federal Canadian prison, we found 41.4% of participants were concerned about the presence of fentanyl, whereas 58.6% were indifferent, a finding we first explore. We tested whether there were any differences between the 41 participants who were concerned about fentanyl and the 58 participants who were indifferent to fentanyl across participant demographic information. The sole demographic variable that was significantly associated with views of fentanyl was age, χ^2^(3, *N* = 99) = 8.425, *p* = .038, Cramer’s *V* = 0.292. Specifically, we found older participants (45–54; 100%) were more likely to report being concerned about fentanyl than younger participants (19–24; 31%) who were less likely to report being concerned about fentanyl. Our qualitative findings also present how, for many officers, fentanyl was one of many normalized risks within the prison environment, including how COs responding to overdoses has become, for the majority of officers, a normalized occupational hazard. We specifically address how COs conducted cell and institutional searches, their vigilance when at work, and views of naloxone administration.

### Fentanyl overdoses

When participants were asked if they had ever responded to an overdose, approximately one third (32.8%) affirmed they had responded to an overdose; in most cases, they reported the occurrence was due to fentanyl, while the majority (67.2%) had not responded to an overdose. We found overdoses did not have the overwhelming impact we anticipated among respondents, as none indicated the presence of any lingering symptoms or signs of mental distress post exposure.

Instead, as per P10 who, when asked about overdoses, said “ah, for me personally, they’re no big deal,” suggests the response is normalized, which is echoed by many. P270, who has responded to multiple overdoses, explains: “I mean it’s nothing crazy. I mean it’s a job. It’s the job I signed up for and that’s what I signed up to do.” P270 describes overdoses as part of the job they “signed up for,” framing their emergency response as an impersonal, occupational responsibility.

Speaking to the ‘business as usual’ aftermath of an overdose response, P373 states “for an overdose situation, generally speaking, no, you’re gonna stay at work and you’re gonna kinda finish your day.” This normalization was further nuanced in P374’s description of their experiences and provides evidence that critical incidents do not affect everyone the same, and even the most extreme cases may be ‘brushed off’ so to speak by officers. This officer explained that in their experiences there have been a “couple of bad overdoses, I worked on a guy for over an hour. He died in front of me multiple times and [I] just keep bringing him back” (P374). Despite the prisoner’s death, when we asked the participant if they were okay afterwards, they explained that they have come to terms with the reality of death. Demonstrating mental health literacy, this officer said “I’d say yeah, just my own mental health and now that is—that’s who I am. So, I’m going to look after me first” (P374). They prioritize self-care and rationalize the event by coming “to terms” with the reality of drug use in prisons, or anywhere for that matter, can result in overdoses and they may be the first responder in such a situation.

This theme was common among participants, as echoed by P393, where an overdose was “my first hands-on incident.” After administering first aid and calling “the appropriate staff,” they explained that when responding, “You just kinda go with it, I guess. You just kind of deal with it as they come, I guess. That’s what I kind of do with work, I just, you never know what might happen [laughing] in your day here, so you just kinda go with whatever comes” (P393). P406 further stated: “there’s not too much I can say about it: you respond, you render [aid] and the job goes on.” Following a response to an overdose, they neither took time off work afterwards nor participated in a Critical Incident Stress Management (CISM) debrief or activity. Regarding CISM, they explained “I think it’s incredibly helpful to have that resource available should staff ever need it. Speaking on a personal level, I didn’t need it” (P406). Not only did P398 voluntarily choose not to be part of CISM following an overdose response, like others, they stated that “nothing was offered after that incident.” P296 too was okay after a close encounter with an overdosing prisoner, but was offered the service nonetheless:

CISM was offered but I didn’t take it because I mean I felt totally fine. It was crazy but once he had started calming down, I guess then I knew that the situation was getting under control and he was coming back to his senses (P296).

Speaking more about CISM, given the fact that CISM was offered regularly to this participant, P373 calls their institution:

*Funny* because lots of times we will get CISM for incidents that nobody’s even really batted an eyelash at and then we’ll have something happen and they either don’t have the staff to provide it or it just kinda gets forgotten about because it’s too busy (P373).

Suggesting overdoses are normalized such that the offering of CISM seems “funny,” but not unnecessary, P373 describes CISM as almost awkward because the critical realities of the incident are not often experienced as such, or at the very least, socially constructed in this way when recounted.

### Concerned about fentanyl

The 41 participants who reported being concerned about fentanyl were adamant in their worries—for these participants, fentanyl created vulnerabilities, including threat to their life and health. P117 said “fentanyl is terrifying,” clarifying they are speaking from “second hand stories from good friends” and without personally interacting with the drug. The source of the concern is multifold. Officers were scared of exposure, concerned about their health and life, and concerned about prisoners overdosing/dying. P104 explained that: “In itself that’s [fentanyl] a big one right there for me. Yeah, fentanyl, like doing cell searching and something or being exposed to that fentanyl and that terrifies me I mean…fentanyl that’s big right now for me.” P104 worries about fentanyl exposure and explains how exposure is possible when conducting their occupational responsibilities. The worry is rooted in the belief that, as per P105, “it’s like one speck of that dust will kill you in a split second.”

While the concern that brief fentanyl exposure on the skin is lethal is not rooted in scientific evidence and has been debunked by several researchers [35, 36], the association between fentanyl and immediate death was often made by participants, such as P143, who shared “we had a tip that there was fentanyl in the institution, so that’s a weapon for me cause a lot of people just by touching it die.” Likewise, P107, who works in an institution that has endured an influx of fentanyl and associated overdoses, explains that fentanyl is a “big time” concern. They felt, given the institutional history, that fentanyl “scares everybody which is fair cause it’s a very dangerous it’s a dangerous substance right,” and that among incarcerated people, “some of them like it.” Here, there appears to be heightened fear around the lethality of the substance, amplified by the perception that some incarcerated people welcome its presence and therefore may commodify it, potentially rendering the prison environment highly volatile and dangerous for the people directly using the substance.

Despite recognizing some incarcerated people want to use fentanyl, officers also felt incarcerated people were concerned about fentanyl, particularly in institutions where fentanyl had resulted in prisoners dying (i.e., overdoses). P13, who works at a prison where there were overdose deaths, explained: “what I was told is Feb 2018 because there were some deaths from it and now the inmates are self-policing for fentanyl because they don’t want to die [from it].” This was echoed by P24 who describes how self-policing presents among incarcerated people: “But the inmate population has said that if they find out guys are bringing in fentanyl then they will like put a stop to it cause they lost a lot of good friends to fentanyl overdoses.”

Some participants in our sample had direct exposure with fentanyl or very close potential exposure/near misses (i.e., a nearby overdose, finding a package, exposure in close proximity). Exposure or near exposure left these officers feeling vulnerable and concerned about fentanyl. In this sense, experience with the drug did affect officers. For instance, P103 describes how, at their prison, they “had a couple lockdowns for fentanyl or whatever [carfentanil].” They, however, take the lockdowns seriously having seen the impacts of fentanyl. They explain how although never directly responding to an incident involving the drug, “I saw a guy on a stretcher the other day… I didn’t directly respond.” Another participant, P113, with direct exposure, explained:

and I’m going in a lot of situations in blind cause yeah we have an inmate that’s non-responsive but I don’t know if it’s a heart attack, I don’t know if it’s a stroke, I don’t know if it’s cause he took fentanyl. I don’t know because there’s fentanyl in the room so it’s again another stressor to the job that as an everyday citizen you don’t even dream of and I could have to respond to four of them tomorrow. I could get a call right now with me on the phone and I’d have to immediately hang up with you and run to wherever it is … fentanyl is still a very big thing within the prison.

P113 describes the vigilance required when fentanyl is present and how the drug affects their decision making and response capacities in emergency situations. Nevertheless, in both cases, the participants have witnessed the impacts of fentanyl on user well-being, even, in the case of P103, without directly responding to such calls for intervention.

### Indifferent to fentanyl

Most COs we interviewed were indifferent to the presence of fentanyl, meaning they were aware of but not fearful of the drug’s presence – thus not concerned about being exposed to fentanyl or the effects of its presence in the prison. As P106 explains:

Everybody is aware of it. But I don’t know that it’s a major fear with staff. I don’t know if it’s something that has many people stressed or like I’m not sure where it would fall on the hierarchy of job concerns. But I don’t feel like for myself that it’s really high up there (P106).

The general awareness, laced with a lack of concern, suggest fentanyl, and the associated risk posed, is normalized and therefore viewed as a routine potential hazard of correctional work. Officers understand that their job entails managing the risk posed by fentanyl. P16 explains the reasons and assumptions surrounding how the presence of drugs is normalized:

I know it here. I’m aware. Like I said, I think people are naïve and stupid if they think drugs aren’t going to be in jail… You just have to be diligent in being careful… It’s the job like that’s the thing it’s what I signed up for. I know what I got (P16).

P16’s words illustrate the “what I signed up for” attitude that underpins, at least in part, the normalization of working in the environment created by the (potential) presence of fentanyl. These officers, however, are unique; they entered the job at CSC after the problem of fentanyl had peaked in federal prisons. They were aware of the risk at occupational entry, and thus how they respond to a risk with pre-emptive knowledge and awareness versus a new and emergent risk may be different.

Some participants felt reassured by their training in wearing personal protective equipment and policy knowledge when navigating the drug’s presence. For example, P149, who is indifferent, is confident in their preventative capacity, explaining they “like policy, I like to read policy so I feel I’m pretty familiar with our highly toxic substance protocol and stuff. So I think if you know what you’re doing and you’re doing it properly, you’re fine.” Evidenced in P149’s words is confidence in how the policies and education will protect them, which is in tension with previous (and scientifically unsupported) beliefs raised by participants about the ‘instantaneous’ lethality of the substance of which a simple lapse in judgment, reaction, or ‘luck’ could result in CO death [35, 36].

Meanwhile, P18, who was also indifferent, when asked if their institution had experienced fentanyl, remarked: “Oh, fuck yeah, it’s everywhere.” However, they continue to explain that they do not feel at risk of intentional exposure as fentanyl is not weaponized, “an inmate’s not gonna use it on you [because] it’s too expensive.” Instead of being fearful, they stated: “I’m not scared, I’m not worried, but I’m cognizant that I know that it is here…” Here, there is a separation between awareness and actual fear of the high presence of fentanyl, and concerns about the substance being weaponized against COs are shrugged off due to the drug being expensive – which is an ironic comment given the participant states that fentanyl can be easily accessed, nonetheless.

P69 also evidences in their words the extent to which the normalization of fentanyl produces indifference toward toxic substances in institutions, explaining:

We still do our jobs normally as if like fentanyl wasn’t introduced at all. We still take the same safety when we do searches and stuff like that, like feathering books away from you and so on and so forth cause it’s a very real possibility that you could get exposed to it but I feel like it’s been around long enough that people kind of know the process [that] you were exposed, if you were to be exposed to it like the process to like get you better. Like obviously, they will give you Narcan so on and so forth to keep you, keep you going until you get medical attention. And everyone knows to like, everybody knows right now if somebody is incapacitated or whatever um or like seizing so on and so forth or if they’re just doing anything abnormal and they’re conscious but not truly there, it’s like ‘alright, we’ll give you Narcan,’ cause Narcan really doesn’t, Narcan isn’t going to kill you… (P69).

P69, in describing the processes around fentanyl exposure, reveals how engrained processes to manage fentanyl exposure are at their institution. They conduct their occupational responsibilities as though “fentanyl wasn’t introduced at all” (P69), they follow their safety training, and they know what will happen and what they are expected to do, if they or another colleague are exposed. In this way, as per P373, “we’re just so used to it now that it’s like just an old hat,” such that “I just kind of assume that it’s a thing for everyone” (P119).

Another theme among many participants who were indifferent was a lack of direct contact with the drug and often a lack of experience. For instance, P108, who is indifferent, explains:

Since I’ve been here, I don’t think we’ve had too many run-ins with fentanyl. I can’t think of any. But I believe maybe a year before I started, there was like a big run of fentanyl, so I mean I guess not too much [of a concern] because it’s not our main concern right now…. *But maybe if you’re having issues with it then it would be you know*, *more on your mind* (P108).

P108 clarifies that perhaps without exposure the drug is less concerning, or the risk and perceived threat posed feels less immediate when the drug is not or less present. This finding was echoed by P114 who likewise appears indifferent to the presence of fentanyl but understands their interpretations are a result of their prison of employment. They say that fentanyl is “not huge, they have found it but it’s not rampant like it is in other institutions that I hear” (P114).

### Impacts on perceptions of occupational responsibilities

Whether concerned or indifferent, fentanyl still affected how officers conducted their occupational responsibilities. Many persons (*n* = 27) who were concerned about fentanyl described how the drug’s presence increases their vigilance. Here, participants explained, due to their concern about fentanyl exposure, they are “more careful but at the same time I’m still super careful with everything I just I treat every inmate like they have [insert infectious disease] in a way, at least I’m making sure that I’m not catching anything” (P105). P105’s words exemplify how the presence of fentanyl is conceived as a constant threat and in consequence they are “more careful” when performing their occupational responsibilities. Revealing the stigma that still surrounds infectious diseases, they explain how they assume all prisoners have contracted some illness and draw on such a belief and assumption to help inform how they practice safety. Through this risk-averse lens, they too recognize that the presence of fentanyl “gets your guard up quite a bit.”

Beyond increased vigilance, if indifferent or concerned, participants explained that the presence of the drug informs how participants perform their occupational responsibilities, such as cell searches, and their views on naloxone, realities to which we now turn.

#### Cell searches, personal protective equipment, and institutional security

The impacts of fentanyl were most pronounced in cell and institutional searches, where the majority of participants (*n* = 50) described taking extra care and precautions to reduce risk of exposure. P12 explains that “It’s just another one of those reality things just if you’re doing searches make sure you’re careful in how you conduct them so that you’re not going to expose yourself,” while P158 notes: “it’s kind of scary to…have to deal with that kind of thing.” P158 explained that at work they are

just more mindful, more cautious and so for example you know, if I’m doing some searching and I’m looking through books or something like my like fan something like that up into my face, I’m really mindful about how I’m handling their belongings.

P12 expresses caution when searching, while P158 describes what caution looks like. Echoing others, they explain that they “fan” books away from their face and are mindful when handling belongings in the belief that even minimal exposure can be life threatening. The concern around fentanyl being hidden in books and belongings was commonplace, as P150 expresses their concern for fentanyl is:

low [but they are] just being mindful when doing room searching to use the appropriate equipment and search the way we were taught at Core. Not opening books right near your face but holding them down and opening them up, face down so that if there is anything it’s not going to spray you in the face, that kind of thing (P150).

P150 draws from their training to ensure they are protecting themselves from exposure. P117 then describes how the presence of fentanyl shapes their occupational work, explaining how this perceived risk impacts their discharge of duties:

Going into a cell you never know what you’re going to see or touch that could potentially harm you. Fortunately, when you go in there’s usually one other person there with you so if something were to happen to you then you have another person who’s watching your back (P117).

P117 explains the value of having a partner when searching, recognizing the interdependence between colleagues for support in the event they are exposed to the substance. For instance, when asked how fentanyl affects how officers do their job, P10 explains that the drug is on their mind “anytime you do a cell search.” P10 continues to describe how the processes around cell searches became even more controlled during times of increased fentanyl use:

…if you and I were doing a cell search, we’d always have somebody else always keep an eye on the cell… We would carry a thing of Narcan in my pocket so I would grab the Narcan in my pocket all of a sudden you open up a drawer and all of a sudden powder goes into your face and then you fall down and I have Narcan in my pocket (P10).

P10, who carries naloxone during cell searches, describes a more controlled search process designed to combat the perceived risk of fentanyl exposure and be immediately responsive in the event of an overdose/exposure. The pairing of officers on searches, although standard practice, increased in importance when confronted with possible fentanyl exposure. Others spoke about the importance of wearing a mask, like P104, who explains “I try to be like a little more like cautious when it comes to like searching or something like to like have a mask. Wear a mask.”

#### Naloxone

Naloxone was often described as instrumental to officers to ensure their safety, such that its presence helped officers manage the stress that accompanies the presence of fentanyl. Lack of access to naloxone was concerning for officers. For instance, P56, describes how fentanyl “is always one of those things that’s always in the back of the mind, I don’t agree with corrections [that] we don’t carry Narcan on us. We have it in our unit pods.” P56 takes issue with the lack of personal access to naloxone, feeling that having it only in the units is insufficient, likely because obtaining the naloxone may create a critical delay in response time as opposed to COs having it on their person.

Curious if such practice was common, we asked P56 (and other participants) as the conversation progressed: “Is that at all institutions? You guys don’t carry Narcan on you?” to which P56 replied:

I believe so, I’m not positive but for sure here because it’s too expensive for them to give them to everybody so they have. I think like four or five in our unit pods. But, yeah, I would rather have it on me (P56).

Thus, they felt hindered by their access to naloxone and preferred to have their own personal use supply, regardless of the costs acquiring it poses to their employer and institution. P154, who works in an institution that has experienced the presence of fentanyl, explains “that’s the majority of the issue in our institution.” They speak to the outcomes of such drugs in prison, noting that.

we should be allowed to have enough Narcan. … We’re doing a walk and then all the sudden what we see an inmate potentially overdosing, it, it hurts, it doesn’t hurt at all to give them Narcan. So if it is a potential overdose, we have that right on our duty belt (P154).

P154 extends the concerns raised earlier by several participants over their personal or colleagues’ health and safety to that of people who are incarcerated – they frame the act of administering naloxone as not “hurting” and requiring as much immediacy as possible to preserve life.

For some, the desire for personal use naloxone was enough to prompt the officers to purchase or acquire their own supply. For instance, P350 said:

I grabbed one [naloxone kit] just in case cause if I was ever washing my clothes at home or anything like that and there was something on that, I just didn’t wanna risk it. I didn’t wanna risk anyone else [coming into contact] so I have something here at home and obviously work has it everywhere so. That was something that came to mind so I thought might as well and it’s just something in the house now (P350).

P350’s words illustrate how they acquired personal use naloxone to offer protection when working and to protect their families and selves at home if accidentally exposed. Overall, naloxone appeared to exist as a remedy for some concern that presents with fentanyl – a reason for officers to be indifferent or accepting of the process of normalization of risk in correctional work. To this point, naloxone seems to have become part of the normalization of fentanyl in prisons; for some, naloxone creates a sense of comfort, like P114 who explains “it’s scary but I guess that’s why we have Narcan. Here it’s not just for inmates, it could be for officers too.” Speaking to normalized processes around naloxone, P114 recognizes naloxone offers reactive protection for incarcerated people and staff alike, and thus some element of comfort while immersed in a work environment laden with concerns around substance (mis)use.

## Discussion and conclusion

In the current study, we sought to determine whether the presence of fentanyl adds to operational stress and affects how COs engage their occupational responsibilities. We have argued participants were both “normalized” to fentanyl as a workplace hazard—as demonstrated through expressions of indifference or lack of concern toward fentanyl and an understanding of fentanyl as present in their institution—as well as deeply troubled by fentanyl as an underpinning social concern. In this context, we invoke the concept of normalization broadly, to refer to social processes wherein ideas, perceptions, and norms become taken-for-granted in everyday life and culture; norms that are dynamic or “actively *shaped* standards for moral evaluation in each group” [54]. For example, as a recreational drug, fentanyl has been found in the United Kingdom to be more socially acceptable and responses in the criminal justice system have begun to consider this cultural reality and complexity [55]. Overall, whatever the source of the indifference among participants, the majority had come to normalize fentanyl as an inherent risk to their occupational work. They did not feel the drug would be weaponized to create a direct threat, but they understood fentanyl posed some risk to correctional work, often which can be managed by adhering to the policies, procedures, and training regimen of their institution.

In this context, nearly a third of COs had been exposed to an overdose during their first or second year on the job; however, largely unexpectedly, most COs who had exposure to an overdose normalized the event. Of note, an overdose constitutes a critical incident requiring post-action care and interventions for people who witness them if understood within a lens of best practice for psychological wellness [56]. Thus, our findings somewhat complicate the narrative of overdosing constituting psychologically traumatic events with causal implications for constituting occupational stress injuries and, potentially, leading to the development of mental health disorders [56]. Future research examining if/how overdoses compare in impact on wellness in perception and response to interpersonal violence among incarcerated people is warranted. However, we emphasize these findings emerge in relation to a sample of federal COs and Canada and do not necessarily represent the reactions and responses of all overdose witnessers.

Another finding was 41.4% of our participants were concerned about fentanyl—a large number but less than we expected—instead, most were indifferent about fentanyl’s presence in prison, with younger COs being less likely to be concerned than older COs. Thus, age, not occupational tenure, affected concern. Many factors could explain this, from the current social climate, to awareness and knowledge, to life experience or education around substance use and overdose. Thus, to explain the findings by age, requires further inquiry.

This indifference to fentanyl among COs could be seen as a positive attribute, as indifference indicates little worry about fentanyl exposure among COs. Future research however warrants an in-depth investigation into if/how the lack of concern is rooted in accurate knowledge or informed by myths about the substance. Further, our findings reveal again [57] how some officers appear to ‘treat lightly’ the potential stressor or trauma they may experience in their duties due to their perceptions of the risk from fentanyl and, likely, the culture of silence that still permeates some correctional workplace cultures. While ideas for change are shaping new trajectories for the mental health treatment and care of correctional workers in Canada [58], more needs to be done to penetrate mental health stigma, as the stoicism resonating in our data may raise doubts about the officers’ willingness to speak in depth about their own stress and mental health issues as they relate to the presence of fentanyl in their workplace.

Whether COs were concerned or indifferent about fentanyl, its presence still affected how they went about their occupational responsibilities, particularly when searching cells or other areas of the institution, their use of personal protective equipment, and in how they understood the value of accessing and administering naloxone. The perceived risk posed by fentanyl then has a wraparound effect, which officers believe poses a threat to family if the substance were to come home with the officer. In essence, COs became more cautious with the presence or potential presence of fentanyl, but their searching activities and vigilant protocols were normalized as necessary with an enhanced importance—the “right way” to do their jobs to prevent harm, as well as naloxone being widely understood as a preventative measure. COs adhered to policies around using personal protective equipment and the skills learned during their training to protect themselves against the perceived threat. Most COs felt the real or imagined fentanyl risk made them behave and act in ‘safer’ ways when doing searches, with the threat having a positive impact on policy adherence and accompanying actions to maintain personal and institutional safety. Ensuring cell searches were conducted in pairs was a safety net for participants in case of an accidental exposure as was the presence and accessibility of naloxone.

COs believe naloxone mitigates the impacts of fentanyl and saves lives. Administering naloxone was never viewed as life threatening – a correct and scientifically sound perspective [59]^1^ – and provides COs and prisoners with a necessary form of protection from exposure, intentional or otherwise. Naloxone was available in their institutions but not carried by each CO personally, which constituted a stress factor for some who worried about accidental exposure. The negative consequence of not carrying naloxone was understood as having possible negative outcomes for COs and prisoners due to the added time to administer naloxone, given it had to be first acquired on a unit and then used distally. COs, we found, would prefer to each have access to naloxone on their person. As another potential way forward, the personal purchase and possession of naloxone is one way officers could mitigate the perceived risk.

One in three COs did respond to overdoses early in their occupational tenure. Overdoses will happen in prison, which has implications for training and mental health awareness and management. As such, CSC must make recruits, during training, aware of the possibility and properly educated on any governing policies around responding to overdoses. While many participants normalized overdose incidents and preparedness, we still recognize that overdoses can constitute a stressful event and thus preparation and mental readiness to respond to these critical incidents is necessary to support resiliency among public safety personnel. Nevertheless, not all COs will feel prepared to respond to overdoses; as such, these officers require training to remain confident in their processes and adherence to policies when engaged in such experiences. Fentanyl made COs more vigilant—if concerned or not, vigilance was one way to avoid threat, but must be paired with appropriate training, resources, and support.

Previously, research has found that COs are concerned about, or fear, fentanyl [32]. We found that, among COs who were concerned about fentanyl, and consistent with Bucerius and Haggerty’s findings [32], some COs feared they or incarcerated people would be intentionally or, most often, accidentally lethally exposed. In their first responder role, COs have a duty to save lives—including when fentanyl is present. When fentanyl is present, the likelihood of COs having to respond to an emergency increases. Emergencies can evoke many outcomes (e.g., investigations, testimony, guilt, liability, physical harm) requiring further exploration. This feeling persisted, despite science and evidence, which both failed to inform interpretations of the lethality of fentanyl exposure.

Interestingly, through informal networks and a kind of conduct code among incarcerated people, COs discussed how incarcerated people expressing and feeling concern about fentanyl responded proactively by working collaboratively toward its elimination, at least in one federal institution where overdoses were plentiful. The very fact that officers recognized these concerns and the work being done to police fentanyl among incarcerated people also lends to the reality that this helps officers do their job— it is *their* responsibility to preserve life and they are liable and held accountable for events on their shift—thus the vigilante efforts of incarcerated people to take their matters of safety into their own hands is appreciated by some officers.

Our data showed overdose response and awareness did not generally have lasting effects on officers. Nevertheless, given one cannot control how an event impacts them, we recommend management consider making sure CISM or effective evidence informed debriefing activities are available after all overdoses/incidents because some may require intervention or benefit from debrief even if they present as unaffected – which is common in the stoic correctional work environment [61]. There appears to be a mix of COs feeling stoic and resilient enough that they can recover from the aftermath of an overdose response without psychological help services, alongside the employer sometimes not offering such services outright, which may be tied to frequency of exposure and the resultant normalization or the correctional culture needing to be ‘okay’ on one’s own following critical incidents [57]. The inconsistent offering of CISM found in our data, needs to be understood in the context of correctional work where overdoses and fentanyl are normalized such that either may not be conceived of requiring CISM in light of other occurrences in the institution. Prison is an environment where mental health concerns are normalized [57] and more challenging realities than overdoses and fentanyl exposure require, in participants’ eyes, more rigid responses. Nevertheless, whether or not CISM (e.g., debriefing) is deemed necessary, our participants did find CISM was at least partially beneficial for some.

Overall, in the current study, we examined the current environment, where fentanyl was slightly displaced from the centre of our attention given the COVID-19 pandemic/endemic, which also affected data collection. In addition, we recognize the degree of presence of fentanyl within each institution may be impacting our results, thus an institutional level analysis would be a welcomed next step to truly differentiate how the drug’s presence impacts officer concern. How drugs enter federal prison is beyond the scope of the current article and an area in need of further inquiry. Additional areas of inquiry include more focused studies with a lens of gender or other socio-demographic variables of officers.

Our data are limited by our sample characteristics, as we only examined the impacts of responding to overdose and fentanyl on officers who possessed one or two years of occupational tenure. The longitudinal data in the current study have yet to be analyzed, and it is important for future studies to consider the perspectives and lived experiences of COs with longer professional experience. That said, clearly, there is misinformation about fentanyl, but the reality remains that COs feel vulnerable given the drug’s presence – thus the situation they define is real in their psychological consequences. With this in mind, we cannot discount the possibility that fentanyl overdoses and the perceived threat of exposure become more pronounced with occupational tenure, but may be countered with awareness and educational programs that correct and meaningfully engage misinformation about fentanyl exposures. Future research should also consider identifying in further depth dissemination patterns of (mis)information about fentanyl, particularly through social media [8].

## Data Availability

The datasets generated and analyzed during the current study are not publicly available due to confidentiality and privacy reasons but are available from the corresponding author on reasonable request.

## Footnotes

1 The risks associated with naloxone administration are low but may include: potential for a needle-stick injury if naloxone is administered intramuscularly; responding to an opioid overdose may evoke a stressful experience; lastly, opioid withdrawal may put the naloxone responder in contact with bodily fluids (i.e., blood, vomit) and potentially cause physical injury [60]. That said, giving people naloxone who are unconscious because of a non-opioid overdose is unlikely to cause harm, and very rarely someone will experience a life-threatening allergic reaction to naloxone [59].

## Abbreviations

CO: Correctional Officer
CSC: Correctional Service Canada
OAT: Opioid Agonist Treatment
CISM: Critical Incident Stress Management
